# Progress in Surgery of the Stomach

**Published:** 1901-01-26

**Authors:** 


### PROGRESS IN SURGERY OF THE STOMACH.

**Endoscopy of the Stomach** by straight and rigid tubes has not met with much success, but Kelling[1] claims to have invented an instrument which, though flexible, can be made straight and which possesses considerable clinical value. The instrument consists principally of a flexible tube connected with a smooth rigid metal tube, which enables the apparatus to be easily moved and rotated betwen the teeth. The flexible portion consists of links fastened together by rivets, and the whole of the flexible portion is covered with an indiarubber tube. The principle followed in changing the flexible tube into a straight, rigid one is taken from the human finger, if it is imagined to be hollow. The extensor tendon is here represented by a wire, traction on which is made by a lever. The lower end of the instrument bears a reflecting prism and an incandescent lamp. Gastroscopy is indicated when there is a well-founded suspicion of the existence of a disease which cannot be cured by internal treatment. The indications are therefore the same as for exploratory laparotomy. It will be of great use in the early diagnosis of carcinoma, in detecting recurrences after operation, in the diagnosis of ulcer, or hour-glass contraction of the stomach, and in ascertaining the presence of foreign bodies.

**Perforating Gastric Ulcer.**—Lund[2] gives statistics to show that the percentage of recoveries after operation for this condition has steadily improved. Amidst cases operated upon days or weeks after perforation an occasional recovery is found, as the ulcer, whether on the anterior or posterior surface of the stomach, sometimes opens into a cavity walled off by adhesions; but the urgency for immediate operation is very plainly demonstrated by these statistics. When we are dealing with a perforated gastric ulcer, the most important point to remember is that the patient's chances of recovery are diminishing at the rate of about 8 per cent. an hour from the occurrence of perforation to the time of operation (unless we are dealing with a case in which protective adhesions are present), so that the indication for immediate radical treatment is peremptory.[3] A series of eleven operations for perforated gastric ulcer is given by Hume,[4] of which five died. In the cases that recovered the interval between perforation and operation was from six to twenty-eight hours. In only one instance was the perforated ulcer found in the posterior wall of the stomach. The prognosis, in these cases, according to Finney,[5] depends largely upon several factors:—1. The condition of the stomach, whether empty or full, when the perforation takes place. 2. The interval between the last meal and the perforation. 3. The time elapsing between the perforation and the operation. 4. The number and size of the perforations. 5. The position of the patient at the time of the perforation. 6. The perfection of the technique at the operation. 7. The nature of the infectious agent, whether streptococcus pyogenes or not.

**Foreign Bodies in the Stomach and Œsophagus.**—Edmunds[6] mentions a successful case of gastrotomy for the removal of a plate of false teeth—consisting of three teeth and three hooks, and measuring about two inches by one inch—which had become impacted in the lower end of the œsophagus. Swift[7] records the case of a child of four years of age who swallowed a whistle which was passed per anum twenty-one days afterwards. The position of the whistle in the stomach could be plainly seen with the X-ray screen. It was round, one inch in diameter, and five-sixteenths of an inch thick at its thickest part, tapering towards its edges.

**Dilatation of the Stomach.**—According to Bidwell[8] the principal causes of this condition are:—Cicatricial contraction of the pylorus, due usually to old gastric ulcer. 2. Malignant disease of the pylorus. 3. Adhesions outside the pylorus. 4. Gastroptosis. 5. Spasm of the pylorus from ulcer, or from excess of free hydrochloric acid. 6. Atony. 7. Various neuroses. 8. Floating kidney on the right side. The surgical procedures which are undertaken for the relief of dilated stomach vary with the cause. In malignant disease they are either pylorectomy or gastro-enterostomy; in non-malignant stenosis they are either pyloroplasty, gastro-enterostomy, Loreta's operation, pylorectomy, or division of adhesions. In gastroptosis and neurotic cases lavage and massage will usually suffice, but in obstinate or long-standing cases, where the dilatation does not yield to these means, the following operations may be undertaken: In gastroptosis the stomach may be stitched to the parietal peritoneum (gastropexy), and in simple dilatation the capacity of the stomach can be diminished by folding in and suturing a portion of the anterior wall of the organ (gastroplication). Cautley[9] describes a case of congenital hypertrophic stenosis of the pylorus in a child aged three months, who died the day after admission to hospital. The two chief theories put forward as to the causation of this disease are as follows:—1. It is due to a simple redundancy of fœtal growth. Nature, in her extreme anxiety to provide an efficient pyloric sphincter, has over-exerted herself and produced too great a quantity of pyloric tissue. 2. Thomson suggests that the essential lesion is not a muscular but a nervous one—a functional disorder of the nerves of the stomach and pylorus leading to ill co-ordinated and therefore antagonistic action of their muscular development. Probably the only chance of permanent benefit lies in operation, and pylorectomy offers the best chance of success.

**Gastro-enterostomy**, says Mayo[10] is the most generally useful operation performed in the stomach; in suitable cases prolonging life and relieving pain in cases of cancer of the pylorus. Two methods of gastro-enterostomy are commonly practised—the simple suture operation and the Murphy button. In performing the operation by either method, it is important that the jejunum should be grasped at its origin and a coil formed about fourteen to sixteen inches in length. At this point the mesentery is also of sufficient length to prevent traction. Care should be taken to have the direction of the peristalsis the same in the stomach and intestine when the anastomosis is effected. There has been considerable discussion as to whether the fistula should be established on the anterior (Wölfler) or posterior wall (Hacker) of the stomach. The latter necessitates an artificial opening into the lesser cavity of the peritoneum to get to the desired part of the posterior wall, and requires a larger incision with longer exposure. There are, however, certain supposed advantages with the posterior operation, but Maydl thinks there is in reality no difference in the results, and as the anterior operation is simpler he prefers it. He tries to get the lower border of the anastomotic opening about one inch above the inferior border of the stomach. A funnel-shaped entrance into the bowel is thus secured, which prevents to a large extent the vicious circle of the biliary and pancreatic juices which has proved so prolific a source of danger to the patient.

[1] Lancet, April 28, 1900, p. 1195. [2] Brit. Med. Jour., Aug. 4, 1900, Ep. No. 83. [3] Med. Rec., Sept. 15, 1900, p. 419. [4] Lancet, Nov. 10, 1900, p. 1345. [5] An. of Surg., July, 1900, p. 12. [6] Brit. Med. Jour., Nov. 17, 1900, p. 1438. [7] Austral. Med. Gaz., Sept. 20, 1900, p. 376. [8] West London Med. Jour., April, 1900, p. 77. [9] Lancet July 28, 1900, p. 256. [10] An. of Surg., August, 1900, p. 269.

